# Retraction: Photo-generated radical mediated molecular luminescence enhancement

**DOI:** 10.1039/d6sc90048h

**Published:** 2026-02-27

**Authors:** Jinming Song, Fengling Zhang, Zhenyi He, Lei Zhou, Jingyu Cao, Tao Li, Xiang Ma

**Affiliations:** a Key Laboratory for Advance Materials and Feringa Noble Prize Scientist Joint Research Centre, Frontiers Science for Materiobiology and Dynamic Chemistry, School of Chemistry and Molecular Engineering, East China University of Science & Technology Meilong Road 130 Shanghai 200237 China maxiang@ecust.edu.cn

## Abstract

Retraction of ‘Photo-generated radical mediated molecular luminescence enhancement’ by Jinming Song *et al.*, *Chem. Sci.*, 2026, https://doi.org/10.1039/d5sc06542a.

The Royal Society of Chemistry, with the agreement of the named authors, hereby wholly retracts this *Chemical Science* article due to concerns with the interpretation of the data and the mechanistic conclusions. After publication, concerns were raised regarding the mechanistic considerations, taking into account the previously reported details of benzil derivatives in the literature.

An assessment by a member of the journal advisory board concurred with the assessment and felt that the conclusions of the work were not sufficiently supported by the experimental results.

Given the significance of these concerns, and to allow time to complete further experiments and data analysis to investigate the mechanism, the authors have requested to retract the article. Jinming Song, Fengling Zhang, Zhenyi He, Lei Zhou, Jingyu Cao, Tao Li and Xiang Ma have agreed to the retraction.

Signed: Jinming Song, Fengling Zhang, Zhenyi He, Lei Zhou, Jingyu Cao, Tao Li and Xiang Ma, 17th February 2026.

Retraction endorsed by Rebecca Campbell, Executive Editor, *Chemical Science*.

